# The Clinicopathological Significance of Basal Markers in Early-Stage Invasive Carcinoma of No Special Type of the Breast

**DOI:** 10.5146/tjpath.2020.01487

**Published:** 2020-09-15

**Authors:** Fikret Dirilenoğlu, Demet Arıkan Etit, Halil Taşkaynatan, Ferhan Elmalı

**Affiliations:** Department of Pathology, Near East University, Faculty of Medicine, Nicosia, Cyprus; Department of Pathology, Katip Celebi University Izmir Ataturk Training and Research Hospital, Izmir, Turkey; Department of Oncology, Katip Celebi University Izmir Ataturk Training and Research Hospital, Izmir, Turkey; Department of Biostatistics, Katip Celebi University Izmir Ataturk Training and Research Hospital, Izmir, Turkey

**Keywords:** Basal marker, Cytokeratin 5/6, EGFR, Early-stage breast cancer, Molecular subtype

## Abstract

***Objective:*** Basal markers [cytokeratin 5/6 (CK5/6) and epidermal growth factor receptor (EGFR)] are used in identifying the basal-like breast carcinoma subtype, which is associated with a poor prognosis. However, the clinicopathological significance in early-stage invasive carcinoma of no special type (IC, NST) has not been well established.

***Material and Method:*** In a five-year period, 133 female patients with early-stage IC, NST with a median follow-up time of 89 months were included. The immunohistochemistry-based molecular subtypes were identified according to ASCO/CAP guidelines in 2013. The cutoff values for basal positivity were determined as 10% for each marker.

***Results:*** Basal positivity was recorded in 83.3% (5/6) of triple-negative breast cancers, 50% (2/4) of HER2-enriched, 18.6% (13/70) of luminal B, and 8.3% of luminal A (4/48) subtype. CK5/6 and EGFR positivity were significantly associated with ER negativity (p < 0.001). EGFR positive cases were significantly associated with PR negativity and HER2 positivity compared to negative cases. However, basal positivity was not associated with the patient outcome (p = 0.006 and p = 0.004, respectively).

***Conclusion:*** Basal positive IC, NSTs were associated with hormone receptor negativity and HER2 overexpression; these patients would therefore be less likely to respond to hormonotherapy and more likely to benefit from anti-HER2 treatment as well as dual-kinase inhibitors. The lack of standardization of the definition of basal marker positivity may contribute to the conflicting results of prognostic studies. Hence, further studies focusing on developing a standard protocol for determining basal marker positivity are needed not only for IC, NST but also for other histological types of breast cancer.

## INTRODUCTION

Staging is a powerful tool for determining the prognosis and treatment choice in breast cancer (1). With the advances in screening and increasing awareness of breast cancer, the majority of tumors are detected in the early stage (2). The more precise estimation of outcome is also dependent on other prognostic and predictive factors, such as histological type, histological grade, and the hormone receptor and HER2 status. Nevertheless, some of the patients with early-stage disease still experience recurrence and metastasis unpredictably, largely due to the biological heterogeneity of the disease (3). An extensive search for new biomarkers therefore persists to improve prognostic and predictive estimates.

As a result of global gene expression profiling studies conducted by Perou and Sorlie, breast carcinomas are divided into five distinct intrinsic molecular profiles with different biological and clinical characteristics: luminal A, luminal B, HER2, basal-like, and normal breast-like (4). Basal-like breast cancer (BLBC) is a subgroup of triple-negative breast cancer (TNBC) that expresses high levels of certain proteins, such as keratins (CK5/6, CK14, CK17), epidermal growth factor receptor (EGFR), c-Kit, and vimentin. All of these BLBC-associated proteins have been proposed as “basal markers” (5). Immunohistochemically, a combination of estrogen receptor (ER) negativity, HER2 negativity, and CK5/6 and/or EGFR positivity has been reported to demonstrate a sensitivity of 76% and specificity of 100% in identifying BLBCs (6). Following this study, CK5/6 and EGFR stains have been commonly used in pathology practice and in research to identify BLBC cases (5,7,8).

Since BLBC has been found to be associated with a poor prognosis, the basal markers used in the identification of this subtype have attracted much interest to reveal their prognostic significance in breast carcinomas. Although some of the studies reported that these markers were associated with a poor patient outcome, others found no correlation (9–11).

In this study, our goal was to determine the clinical and pathological value of basal positivity (CK5/6 and/or EGFR), specifically in a subset of patients with early-stage invasive carcinoma of no special type of the breast (IC, NST).

## MATERIALS and METHODS

Our study was approved by our institution’s Non-interventional Ethics Committee with decision number 92 dated 24 August 2016. Informed consent was obtained from each patient included in this manuscript. We performed this study according to the principles of the ethical guidelines established in the World Medical Association’s Declaration of Helsinki.

### Patients and Clinical Information

Between January 2007 and October 2011, female patients with a diagnosis of early-stage (stage I, II, IIIA) IC, NST of the breast were retrospectively analyzed from the electronic database systems of the Department of Pathology and Oncology (Probel Software, Izmir, Turkey) (12). Age, menopausal status, tumor location, type of surgery, number of tumor foci, histological grade, presence of ductal carcinoma in situ (DCIS) or lobular carcinoma in situ (LCIS), status of surgical margins, stage of the disease, status of axillary lymph nodes, immunohistochemical (IHC) stains (ER, PR, HER2, and Ki-67), treatments received [hormonotherapy (HT), chemotherapy (CT), radiotherapy (RT), and other targeted treatment agents such as trastuzumab)], and the clinical follow-up and survival data were documented for each case. Menopausal status was recorded as premenopausal and postmenopausal. The types of operation were breast conservative surgery, simple mastectomy, and modified radical mastectomy. The number of tumor foci was divided into single or multiple. The lymph node status was divided into three groups as N0, N1, N2. The cases with accompanying fatal disease were not included in the study. The cases who did not have sufficient clinical and follow-up data or pathology material of sufficient quality and quantity were excluded from the study.

### Re-Assessment of Histopathological and IHC Characteristics of Tumors

All the slides of the cases with sufficient clinical information were obtained from the archives of the Department of Pathology. Two pathologists (FD and DAE) reviewed all the H&E and IHC slides. The histological grade of the tumors was determined according to the Modified Scarff-Bloom-Richardson system.

For ER and PR, 1% or more staining was considered positive. In assessing HER2 status, IHC and fluorescence in situ hybridization (FISH) analyses were performed according to the updated American Society of Clinical Oncology/College of American Pathologists guideline in 2013. The Ki-67 proliferation index was assessed using a 40X objective lens in the highest area of staining (hot spot).

The cases with HER2 score 2+ were tested by FISH analysis and recorded as HER2 positive or negative. At least 50 cells were counted in FISH analysis and the cases with HER2 signal/CEP17 (chromosome 17 centromere) signal ratio of >2 were determined as HER2 positive.

### Technical Properties of the IHC Studies

According to standard tissue processing and staining procedures in our laboratory, all the specimens were fixed in 10% neutral-buffered formaldehyde solution for 24-48 hours. Tissue samples were processed in an automated closed-system tissue processor and embedded in paraffin. Four-micron sections from the prepared paraffin blocks were mounted on poly-L-lysin slides. For CK5/6 and EGFR, one formalin-fixed, paraffin-embedded (FFPE) block containing sufficient tumor tissue was selected from each case during the histopathological review. Two sections were obtained from each of the selected blocks and transferred on two separate poly-L-lysin slides. The staining procedure was carried out according to the manufacturer’s instructions. The antibodies were visualized by the streptavidin-biotin-peroxidase method using ER (Novocastra, Leica Biosystems, Wetzlar, Germany; mouse monoclonal antibody, SP1 clone, 1:40 dilution), PR (Novocastra, mouse monoclonal antibody, SP2 clone, 1:100 dilution), HER2 (Novocastra, mouse monoclonal antibody, CB11 clone, EGFR (Novocastra, mouse monoclonal antibody, EGFR.25 clone, 1: 100 dilution), CK5/6 (Dako, mouse monoclonal antibody, D5/16 B4 clone, ready-to-use), and Ki-67 (Novocastra, mouse monoclonal antibody, MIB1 clone, 1:100 dilution). For positive controls, normal breast parenchyma adjacent to tumor was used for ER, PR, and CK5/6. An additional section from breast cancer tissues that was positive for the respective stains was used for each of HER2, Ki-67 and EGFR.

**Identification of IHC-Based Molecular Subtypes**

All cases were divided into subtypes consistent with intrinsic breast carcinoma subtypes as outlined in the results of the International Breast Cancer Conference in St. Gallen in 2013.

### Evaluation of CK5/6 and EGFR Staining

Percentage of cytoplasmic and/or membranous staining in invasive tumor cells was recorded. The cutoff values were determined as 10% for both stains. The cases with at least one marker positivity were designated as “basal positive”.

### Data Analysis

All statistical analyses were performed using the IBM SPSS Statistics 22.0 package program (IBM Corp., Armonk, New York, USA). The frequencies of clinical and histological variables were presented using cross-tabulations. A two-sided Fisher’s Chi-Square exact test for *rxc* tables was applied to compare the differences between the groups for categorical variables. The normal distribution of variables was examined visually (histogram and probability plots) and with analytical methods (Shapiro-Wilk tests). If the distribution was not normal or there were ordinal variables, the groups were compared by using the Mann-Whitney *U* test. If at least one of the variables was not normally distributed or ordinal, the correlation coefficients and statistical significance were calculated by the Spearman test for inter-variable relationships. Kaplan-Meier analysis was used for survival analysis and the log-rank test was used for comparison of the survival curves. A value of *p* <0.05 was considered significant.

## RESULTS

### Patients and Clinical Information

One hundred and eighty cases diagnosed as early-stage IC, NST between October 2007 and October 2011 were re-evaluated. Twenty-seven cases without available FFPE blocks (consultation cases), and 20 cases with histologically inadequate quality and quantity of tissue and/or with no invasive tumor area after the sections for CK5/6 and EGFR stains were excluded from the series. A total of 133 cases in which at least one basal marker staining could be evaluated were included in the study.

All cases were female and the *median* age was 50 (range, 33 - 77). Seventy-four (55.6%) of the cases were premenopausal and 59 (44.4%) were postmenopausal. Fifty-seven (43.2%) tumors were located in the right breast, 75 (56.8%) were in the left breast, and the location was unknown in one case. Breast conservative surgery was performed in 79 cases (65.3%), MRM in 38 cases (31.4%), and simple mastectomy in four cases (3.3%). The type of operation was not known in 12 cases. Of the 131 tumors, 116 (88.5%) were in a single focus and 15 (11.5%) were in multiple foci. The *median* tumor size was 2 cm (range, 0.6 - 7 cm). Fifty-eight (43.6%) were stage I, 57 (42.9%) were stage II, and 18 (13.5%) were stage IIIA. Axillary lymph node metastasis was present in 57 (42.9%) of the cases: N1 in 40 (30.1%) and N2 in 17 (12.8%). One hundred and twenty-one cases (91%) received HT, 115 (86.5%) received CT, and 107 cases (80.5%) received RT. *Median* follow-up time was 89 months (range, 27 - 117 months). During follow-up, progression occurred in 16 (12%) of the patients while 8 patients (7.1%) deceased. The *median* progression-free survival (PFS) time was 88 months (range, 14 - 117 months) and the *median* overall survival (OS) time was 92 months (range, 27-117 months).

### Histopathological Analyses and IHC-Based Molecular Subtypes

Among 130 cases, histological grade was grade 1 in 17 (13.1%), grade 2 in 95 (73.1%), and grade 3 in 18 (%13.8) cases. Of 128 cases, 42 (32.8%) had DCIS and one of these cases was found to have LCIS. Among 125 cases, surgical margin positivity was found in three (2.4%). According to the results of ER, PR, HER2, Ki-67, CK5/6, and EGFR staining, 49 cases (35.8%) were classified as luminal A, 48 (36.9%) were luminal B-HER2 (-), 23 (17.7%) were luminal B-HER2 (+) [19 (14.6%) were luminal B-HER2 PR (+), four (3.1%) were luminal B-HER2 PR (-)], four (3.1%) were HER2-enriched, and six (4.4%) were triple-negative breast carcinoma. All triple-negative cases included in this study were classified as BLBC because they showed at least one basal marker positivity. Three tumors with an “ER (-), PR (+), and HER2 (-)” profile were unclassified.

### Staining Properties of CK5/6 and EGFR

Both stains were interpretable in 131 cases out of 133 cases. Due to the technical issues, two cases with CK5/6 and one case with EGFR stain could not be evaluated.

**Figure 1:**
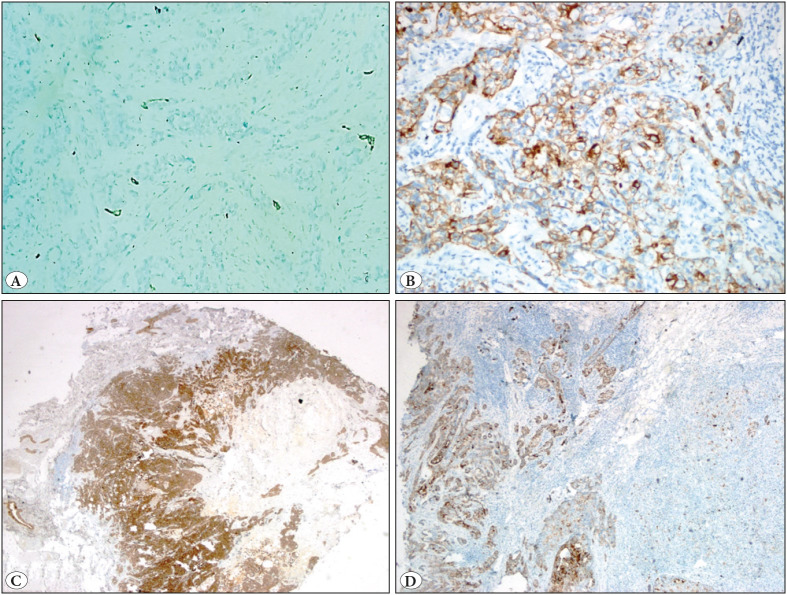
CK5/6 positivity in invasive breast carcinoma, no special type. **A)** Isolated cell staining (IHC; x200). **B)** Focal, membranouspredominant staining. (IHC; x200). **C)** Diffuse staining (IHC; x40). **D)** Mixed staining pattern, areas of extensive cytoplasmic - membranous staining and isolated cell staining (IHC; x40).

CK5/6 showed staining in basal and/or luminal epithelial cells in the normal breast parenchyma. Cytoplasmic and/or membranous staining was observed. Among 131 cases, 9 (6.9%) were CK5/6 positive. When *any positivity* in invasive tumor cells was recorded, 76 cases (58%) were positive for CK5/6. The percentage of staining of the tumors varied between 0 and 100% (Figure 1).

**Figure 2:**
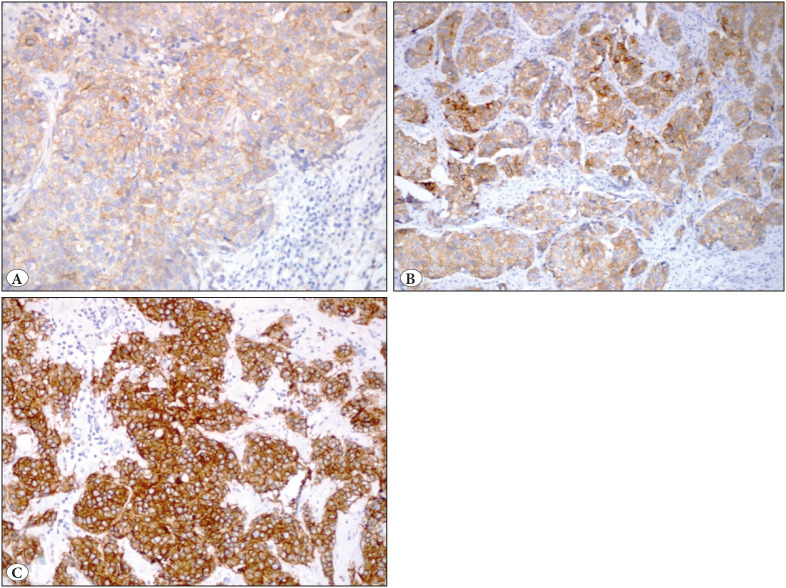
EGFR staining in various intensities in invasive breast carcinoma, no special type. **A)** (1+) intensity (IHC; x200). **B)** (2+) intensity (IHC; x100). **C)** (3+) intensity (IHC; x200).

EGFR positivity was generally weak in epithelial and myoepithelial cells in the normal breast although rarely it was strong. Positive cases had cytoplasmic and/or membranous staining in various intensities (Figure 2). EGFR was positive in 26 (19.7%) of 132 cases. When *any positivity* in invasive tumor cells was included, 69 cases (52.3%) were positive for EGFR.

The correlation between CK5/6 and EGFR positivity was moderate (*rho* = 0.559; *p *< 0.001). However, significant differences between staining percentages were noted in some cases in which both markers were positive. The cases that showed positivity with CK5/6 (n=9) were also found to be positive with EGFR, but not vice versa. The number of basal positive (CK5/6 and/or EGFR positive) cases was therefore the same as the EGFR positive cases. When *any staining* in invasive tumor cells was taken into account, 93 cases (69.9%) were basal positive and both stains were found to be positive in 52 cases (40%).

### The Relationship Between the Basal Marker Positivity and the Clinical and Pathological Data

Each basal marker was correlated with the clinical and pathological data. All CK5/6 positive cases were also positive for EGFR, but not vice versa, and concordant statistical results were therefore obtained in the analyses for EGFR and basal positivity. CK5/6 positivity and EGFR positivity were found to have a significant association with histological grade (*p* = 0.001 and *p* = 0.008, respectively).

CK5/6 and EGFR were significantly associated with IHC-based subtypes (*p* = 0.037 and *p* < 0.001, respectively). Basal positivity was recorded in 83.3% (5/6) of TNBC, 50% (2/4) of HER2-enriched, 18.6% (13/70) of luminal B, and 8.3% of luminal A (4/48) subtype. CK5/6 and EGFR positivity were significantly associated with ER negativity (ER-negative vs. ER-positive cases; for CK5/6, 35.3% vs. 2.6%; for EGFR, 77.8% vs. 10.5%, *p* < 0.001). EGFR positive cases were significantly associated with PR negativity and HER2 positivity (47.1% vs. 15.7% in PR negative vs. PR positive cases, *p* = 0.006; and 14.3% vs. 40.7% in HER2 negative vs. HER2 positive cases, *p* = 0.004) compared to negative cases. Although not statistically significant, CK5/6 positive cases were also more likely to be PR negative and HER2 positive (18.8% vs. 5.2% in PR negative vs. PR positive cases, *p* = 0.08; and 4.8% vs. 14.8% in HER2 negative vs. HER2 positive cases, *p* = 0.086) (Table 1).

**Table 1:** The relationship between basal markers and clinical and pathological data.

		**CK5/6 positivity**					**EGFR positivity**					**Basal positivity**				
		**Negative**		**Positive**		***p***	**Negative**		**Positive**		***p***	**Negative**		**Positive**		***p***
		**n**	**%**	**n**	**%**		**n**	**%**	**n**	**%**		**n**	**%**	**n**	**%**	
**Age**	<40	18	(14.8)	0	(0)	0.351	13	(12.3)	5	(19.2)	0.495	13	(12.4)	5	(19.2)	0.471
	40-55	55	(45.1)	6	(66.7)		49	(46.2)	13	(50)		48	(45.7)	13	(50)	
	>55	49	(40.2)	3	(33.3)		44	(41.5)	8	(30.8)		44	(41.9)	8	(30.8)	
**Menopausal status**	Premenopausal	66	(54.1)	6	(66.7)	0.513	55	(51.9)	19	(73.1)	0.077	54	(51.4)	19	(73.1)	0.051
	Postmenopausal	56	(45.9)	3	(33.3)		51	(48.1)	7	(26.9)		51	(48.6)	7	(26.9)	
**Location**	Right	51	(42.1)	5	(55.6)	0.498	46	(43.8)	11	(42.3)	1.000	45	(43.3)	11	(42.3)	1.000
	Left	70	(57.9)	4	(44.4)		59	(56.2)	15	(57.7)		59	(56.7)	15	(57.7)	
**Operation**	BCS	71	(64.5)	7	(77.8)	0.794	62	(64.6)	17	(70.8)	0.640	61	(64.2)	17	(70.8)	0.639
	SM	4	(3.6)	0	(0)		4	(4.2)	0	(0)		4	(4.2)	0	(0)	
	MRM	35	(31.8)	2	(22.2)		30	(31.3)	7	(29.2)		30	(31.6)	7	(29.2)	
**Number of foci**	Single	106	(88.3)	9	(100)	0.596	94	(89.5)	22	(88)	1.000	94	(90.4)	22	(88)	1.000
	Multiple	14	(11.7)	0	(0)		11	(10.5)	3	(12)		10	(9.6)	3	(12)	
**Size**	<2 cm	73	(59.8)	5	(55.6)	0.583	67	(63.2)	12	(46.2)	0.277	66	(62.9)	12	(46.2)	0.278
	2-5 cm	40	(32.8)	4	(44.4)		32	(30.2)	12	(46.2)		32	(30.5)	12	(46.2)	
	>5 cm	9	(7.4)	0	(0)		7	(6.6)	2	(7.7)		7	(6.7)	2	(7.7)	
**Grade**	Grade 1	15	(12.5)	1	(12.5)	**0.001**	16	(15.2)	1	(4.2)	**0.008**	15	(14.4)	1	(4.2)	**0.007**
	Grade 2	93	(77.5)	2	(25)		79	(75.2)	15	(62.5)		79	(76)	15	(62.5)	
	Grade 3	12	(10)	5	(62.5)		10	(9.5)	8	(33.3)		10	(9.6)	8	(33.3)	
**DCIS**	No	80	(68.4)	5	(55.6)	0.471	70	(68)	15	(62.5)	0.635	70	(68.6)	15	(62.5)	0.630
	Yes	37	(31.6)	4	(44.4)		33	(32)	9	(37.5)		32	(31.4)	9	(37.5)	
**Margin**	Negative	111	(97.4)	9	(100)	1.000	98	(97)	23	(100.0)	0.625	97	(97)	23	(100)	0.624
	Positive	3	(2.6)	0	(0)		3	(3)	0	(0)		3	(3)	0	(0)	
**Stage**	Stage 1	53	(43.4)	4	(44.4)	0.579	48	(45.3)	10	(38.5)	0.755	47	(44.8)	10	(38.5)	0.792
	Stage 2	52	(42.6)	5	(55.6)		44	(41.5)	13	(50)		44	(41.9)	13	(50)	
	Stage 3A	17	(13.9)	0	(0)		14	(13.2)	3	(11.5)		14	(13.3)	3	(11.5)	
**LN stage**	Stage 0	68	(55.7)	7	(77.8)	0.369	60	(56.6)	16	(61.5)	0.949	59	(56.2)	16	(61.5)	0.949
	Stage 1	38	(31.1)	2	(22.2)		33	(31.1)	7	(26.9)		33	(31.4)	7	(26.9)	
	Stage 2	16	(13.1)	0	(0)		13	(12.3)	3	(11.5)		13	(12.4)	3	(11.5)	
**LN positivity**	No	68	(55.7)	7	(77.8)	0.299	60	(56.6)	16	(61.5)	0.666	59	(56.2)	16	(61.5)	0.664
	Yes	54	(44.3)	2	(22.2)		46	(43.4)	10	(38.5)		46	(43.8)	10	(38.5)	
**HT**	No	9	(7.4)	2	(22.2)	0.167	5	(4.7)	7	(26.9)	**0.002**	5	(4.8)	7	(26.9)	**0.002**
	Yes	113	(92.6)	7	(77.8)		101	(95.3)	19	(73.1)		100	(95.2)	19	(73.1)	
**CT**	No	17	(13.9)	1	(11.1)	1.000	16	(15.1)	2	(7.7)	0.372	16	(15.2)	2	(7.7)	0.370
	Yes	105	(86.1)	8	(88.9)		90	(84.9)	24	(92.3)		89	(84.8)	24	(92.3)	
**RT**	No	25	(20.5)	1	(11.1)	0.687	23	(21.7)	3	(11.5)	0.287	23	(21.9)	3	(11.5)	0.183
	Yes	97	(79.5)	8	(88.9)		83	(78.3)	23	(88.5)		82	(78.1)	23	(88.5)	
**ER status**	Negative	11	(9)	6	(66.7)	**<0.001**	4	(3.8)	14	(53.8)	**<0.001**	4	(3.8)	14	(53.8)	**<0.001**
	Positive	111	(91)	3	(33.3)		102	(96.2)	12	(46.2)		101	(96.2)	12	(46.2)	
**PR status**	Negative	13	(10.7)	3	(33.3)	0.08	9	(8.5)	8	(30.8)	**0.006**	9	(8.6)	8	(30.8)	**0.006**
	Positive	109	(89.3)	6	(66.7)		97	(91.5)	18	(69.2)		96	(91.4)	18	(69.2)	
**HER2 status**	Negative	99	(81.1)	5	(55.6)	0.086	90	(84.9)	15	(57.7)	**0.004**	89	(84.8)	15	(57.7)	**0.004**
	Positive	23	(18.9)	4	(44.4)		16	(15.1)	11	(42.3)		16	(15.2)	11	(42.3)	
**Progression**	No	108	(88.5)	8	(88.9)	1.000	96	(90.6)	21	(80.8)	0.174	95	(90.5)	21	(80.8)	0.177
	Yes	14	(11.5)	1	(11.1)		10	(9.4)	5	(19.2)		10	(9.5)	5	(19.2)	
**Death**	No	115	(94.3)	9	(100)	1.000	101	(95.3)	23	(88.5)	0.355	100	(95.2)	23	(88.5)	0.356
	Yes	7	(5.7)	0	(0)		5	(4.7)	3	(11.5)		5	(4.8)	3	(11.5)	

**BCS:** Breast conservative surgery, **SM:** Simple mastectomy, **MRM:** Modified radical mastectomy, **DCIS:** Ductal carcinoma in situ, **LN:** Lymph node, **HT:** Hormonotherapy, **CT:** Chemotherapy, **RT:** Radiotherapy.

### Survival Analyses

Univariate analyses showed that tumor size (*p* = 0.003), surgical margin positivity (*p* = 0.011), stage (*p* < 0.001), lymph node stage (*p* < 0.001), IHC-based molecular subtypes (*p* = 0.002), and ER status (*p* = 0.045) were significantly associated with the OS. Menopausal status (*p* = 0.031), type of operation (*p* = 0.017), increase in tumor size (*p* < 0.001), surgical margin status (*p* = 0.046), increase in stage (*p* < 0.001), lymph node involvement (*p* = 0.009), increase in lymph node stage (*p* < 0.001), and IHC-based molecular subtypes (*p* = 0.007) were significantly associated with the PFS. Log-rank test results for all the clinical and pathological features are presented in Table 2.

**Table 2:** Log-rank test results for clinicopathological features.

**Clinicopathological Feature**		**Overall Survival**			**Progression-free Survival**		
			**Log-rank statistics**			**Log-rank statistics**	
		***mean*** **±** ***sem***	**χ2**	***p***	***mean*** **±** ***sem***	**χ2**	***p***
Age	<40	113.68±4.23	0.909	0.635	104.94±6.48	2.947	0.229
	40-55	116.17±2.18			105.64±3.43		
	>55	115.71±1.85			109.73±2.96		
Menopausal status	Premenopausal	115.17±2.13	0.173	0.678	103.77±3.44	4.658	**0.031**
	Postmenopausal	113.25±1.40			109.14±2.25		
Location	Right	116.54±2.02	0.108	0.743	104.82±3.59	2.625	0.105
	Left	115.54±1.94			110.03±2.79		
Operation	BCS	117.50±1.05	4.545	0.085	111.14±1.92	7.580	**0.017**
	SM	-			-		
	MRM	110.97±3.46			97.06±2.24		
Size	<2 cm	117.37±1.51	11.354	**0.003**	111.96±2.25	21.12	**<0.001**
	2-5 cm	115.99±2.24			104.43±3.43		
	>5 cm	93.70±7.46			71.90±11.48		
Grade	Grade 1	-	0.016	0.613	113.50±3.20	0.542	0.763
	Grade 2	116.96±1.33			108.59±2.46		
	Grade 3	112.67±3.24			104.06±5.99		
DCIS	No	115.88±1.54	0.239	0.625	107.89±2.64	0.047	0.828
	Yes	110.73±1.85			104.01±3.34		
Margin	Negative	116.33±1.19	6.457	**0.011**	108.63±2.09	3.999	**0.046**
	Positive	69.30±1.36			51.67±13.34		
Stage	Stage 1	117.69±1.60	18.891	**<0.001**	114.45±1.82	28.250	**<0.001**
	Stage 2	118.261±0.73			109.55±2.59		
	Stage 3A	95.50±7.25			76.71±9.56		
LN stage	Stage 0	117.64±1.33	11.881	<**0.001**	113.16±1.89	30.651	**<0.001**
	Stage 1	-			109.97±3.01		
	Stage 2	114.2±1.2			74.47±9.91		
LN positivity	No	117.64±1.33	1.17	0.28	113.16±1.89	6.861	**0.009**
	Yes	112.72±2.74			100.33±4.25		
HT	No	106.92±8.62	3.045	0.081	95.17±10.97	2.352	0.125
	Yes	116.91±1.25			109.08±2.15		
RT	No	111.28±1.67	0.264	0.607	106.34±2.48	0.525	0.469
	Yes	115.52±1.67			106.70±2.64		
Molecular subtypes	Luminal A	-	4.946	**0.002**	110.68±2.74	11.232	**0.007**
	Luminal B	113.93±2.02			106.55±2.93		
	HER2-enriched	-			-		
	TNBC	93.83±15.49			73.33±17.95		
ER status	Negative	108.87±6.18	4.006	**0.045**	99.22±8	1.812	0.178
	Positive	117.18±1.25			109.10±2.22		
HER2 status	Negative	116.08±1.59	0.112	0.738	107.79±2.47	0.018	0.894
	Positive	114.69±2.93			107.84±4.88		

**BCS:** Breast conservative surgery, **SM:** Simple mastectomy, **MRM:** Modified radical mastectomy, **DCIS:** Ductal carcinoma in situ, **LN:** Lymph node, **HT:** Hormonotherapy, **RT:** Radiotherapy, **TNBC:** Triple-negative breast cancer, **sem:** Standard error of mean.

**Figure 3:**
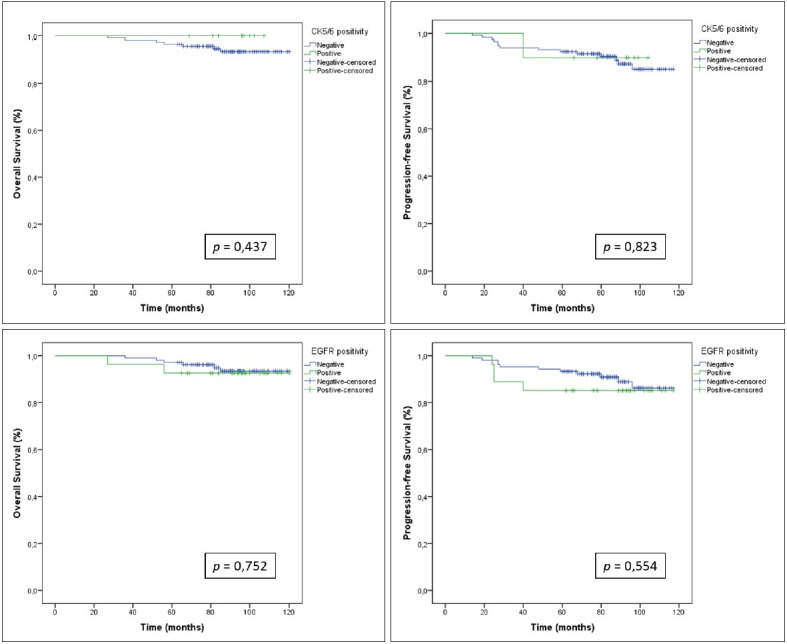
These diagrams display Kaplan-Meier survival curves comparing overall survival and progression-free survival between basal marker positive and negative cases.

Because all CK5/6 positive cases were also found to be positive for EGFR, outcome analyses for the cases with EGFR positivity yielded results that were concordant with those from cases with basal positivity (CK5/6 and/or EGFR positivity). In univariate analyses, EGFR positive cases compared to negative cases showed slightly poorer OS and PFS but this association was not statistically significant (*p* > 0.05). CK5/6 positivity was not associated with the OS and PFS as well (*p* > 0.05). However, CK5/6 positive cases showed slightly better OS compared to the negative cases (Figure 3). Notably, CK5/6 was negative in all cases who died of the disease, and in all but one case who had progressive disease. EGFR was negative in five out of eight cases who died of the disease and 10 out of 15 cases who had progressive disease.

## DISCUSSION

Our study focused on the clinical and pathological utility of the basal markers in early-stage IC, NST of the breast. Our results suggested that basal positive cases, as compared to negative cases, may differ from each other in terms of treatment choice and therapy resistance because they significantly tend to be hormone receptor negative and HER2 positive. Basal positivity did not correlate with the significant prognostic factors, i.e., TNM stage, tumor size, and nodal status (13). Accordingly, basal positivity was not found to be associated with the patient outcome in our study.

Regardless of cutoff value selection, basal positivity (positivity for CK5/6 and/or EGFR) has been reported in 15.6% of all invasive breast carcinomas on average (6,14–16). Among the subtypes, these markers were found to be expressed most frequently in TNBC (50-80%) (14) . HER2-enriched is the other subtype with remarkable basal positivity rates, and EGFR (HER1) has shown positivity in up to 58% in particular (16). It was also reported that basal positivity is significantly more common in HER2-positive compared to HER2-negative breast cancers overall (16,17). However, basal positivity was found to be associated with HER2 negativity in ER-negative tumors (18,19). In accordance with these findings, our results supported that basal markers, especially EGFR, are most frequently found to be positive in TNBC, but are also associated with HER2 positivity in non-TNBC. Besides, basal positive breast carcinomas are more likely to be hormone receptor negative. Considering these associations, these patients would less likely respond to HT but more likely benefit from anti-HER2 therapy as well as dual-kinase inhibitors (such as lapatinib), which target both EGFR and erb-B2 and are promising in HER2- and EGFR-expressing breast carcinomas (20,21). In hormone receptor positive subtypes (luminal A and luminal B), the basal positivity rate was lower (17/118, 14.4%) and this is compatible with the previous studies with rates ranging from 1% to 18% (15,16,22).

Prognostic use of these markers has yet to be established since there is a lack of standardization in defining basal marker positivity. Some of the previous studies have used one basal marker only, while others have used more than one marker. The variations in the types, clones, and evaluation methods of basal markers have been confusing (5,19). For instance, cutoff value selection has varied significantly in prognostic and predictive studies. Some studies that investigated the prognostic and predictive value of basal markers in breast carcinomas have regarded any weak cytoplasmic or membranous staining as positive, while some others determined positivity based on the intensity of staining or a cutoff value of up to 10% (6,11,15). We used the cutoff value of 10% for both markers instead of any cell staining basically for two reasons: i) lower cutoff values showed poor reproducibility in interpretation; *ii)* basal markers are also positive in other non-invasive lesions, such as DCIS, or normal parenchyma. We often encountered weak staining in a very small number of cells within the tumor area and were then unable to discriminate whether the staining was present in invasive tumor cells or not (23,24). Using this cutoff value, 19.7% of the cases were EGFR positive and 6.9% were CK5/6 positive. However, when any invasive tumor cell staining was counted, the positivity rates increased to 52.3% for EGFR and 58% for CK5/6. Another issue is the variation in cutoff values for predictive breast cancer markers. For instance, the cutoff value for ER positivity was determined as 5% in one study and 10% in another (25,26). In our study, we used a cutoff value for ER of 1%, as currently recommended by the ASCO/CAP guidelines (27). The cutoff value for the Ki-67 proliferation index in the distinction of the luminal B from the luminal A subtype is 14% for some authors and 20% for the others (28). In this case, the distribution and clinical characteristics of these groups would vary from one study to another. These observations may explain the disparities between the rates of basal marker positivity and the differences in prognostic estimates reported in breast carcinomas.

We aimed to evaluate the utility of basal markers in early-stage invasive breast carcinoma cases with a diagnosis of IC, NST. Basal positive IC, NSTs were associated with hormone receptor negativity and HER2 overexpression; therefore, these patients may not benefit from HT but may respond to anti-HER2 treatment as well as dual-kinase inhibitors, such as lapatinib. In our study population including the most common histological type of breast cancer and with a median follow-up time of 89 months, previously established strong prognostic factors remained significant. However, basal positivity was not associated with the patient outcome. The lack of standardization of the definition of basal marker positivity may contribute to the conflicting results of prognostic studies. Hence, further studies focusing on establishing a standard protocol for determining basal marker positivity is needed not only for IC, NST but also for other histological types of breast cancer.

## FUNDING

This research paper was generated from the thesis of Dr. Fikret Dirilenoglu and received a grant from the Board of Scientific Research of the Izmir Ataturk Training and Research Hospital (Grant No: 6/2016).

## Conflict of Interest

The authors declare no conflict of interest.

## ACKNOWLEDGEMENTS

We would like to thank Dr. Yuksel Kucukzeybek, Ayhan Kancar, and Hulya Goren for their generous time and great technical assistance.
